# Effect of Electroacupuncture on Pain Perception and Pain-Related Affection: Dissociation or Interaction Based on the Anterior Cingulate Cortex and S1

**DOI:** 10.1155/2020/8865096

**Published:** 2020-10-13

**Authors:** Yan Shi, Shujing Yao, Zui Shen, Lijiao She, Yingling Xu, Boyi Liu, Yi Liang, Yongliang Jiang, Jing Sun, Yuanyuan Wu, Junying Du, Yilin Zhu, Zemin Wu, Jianqiao Fang, Xiaomei Shao

**Affiliations:** ^1^Department of Neurobiology and Acupuncture Research, The Third Clinical College, Zhejiang Chinese Medical University, Key Laboratory of Acupuncture and Neurology of Zhejiang Province, Hangzhou 310053, China; ^2^Department of Acupuncture and Massage, Affiliated Hangzhou First People's Hospital, Zhejiang University School of Medicine, Hangzhou 310006, China; ^3^Department of Rehabilitation Medicine, Changxing People's Hospital, Huzhou 313100, China

## Abstract

Electroacupuncture (EA) can effectively modulate pain perception and pain-related negative affect; however, we do not know whether the effect of EA on sensation and affect is parallel, or dissociated, interactional. In this study, we observed the effects of the anterior cingulate cortex (ACC) lesion and the primary somatosensory cortex (S1) activation on pain perception, pain-related affection, and neural oscillation in S1. ACC lesions did not affect pain perception but relieved pain-paired aversion. S1 activation increased pain perception and anxious behavior. EA can mitigate pain perception regardless of whether there is an ACC lesion. Chronic pain may increase the delta and theta band oscillatory activity in the S1 brain region and decrease the oscillatory activity in the alpha, beta, and gamma bands. EA intervention may inhibit the oscillatory activity of the alpha and beta bands. These results suggest that EA may mitigate chronic pain by relieving pain perception and reducing pain-related affection through different mechanisms. This evidence builds upon findings from previous studies of chronic pain and EA treatment.

## 1. Introduction

For a long time, it was generally believed that pain perception was well understood, while the pain related affection was yet unclear. Recently, the dissociation theory of pain sensation and affection was put forward [1, 2]. Many studies have indicated that chronic pain not only aggravates pain perception but also induces negative affective states (e.g., aversiveness, anxiety, depression, and anhedonia), sleep disorders, abnormal decision-making, and even suicide [3–7]. Approximately 20-30% of chronic pain patients have a negative affect [8, 9]. Pain and related affection influence each other and demonstrate reciprocal causation. Experimental studies have indicated that emotional interventions, such as meditation, not only alleviate pain perception and negative affection but also have a beneficial protective effect on the brain's gray matter and pain regulation pathways [10, 11]. Mitigating chronic pain by modulating negative affect is a new research direction, and the mechanism of the interaction between chronic pain and negative affect remains unclear.

Pain processing involves multiple cortices. In the imaging studies of chronic pain in the human brain, researchers have found that the most frequently activated brain regions include the anterior cingulate cortex (ACC), the primary somatosensory cortex (S1), insula cortex, prefrontal cortex, and thalamus, among others [12]. Most reports have made it clear that the ACC plays a key role in pain-related emotion. Some studies have shown that the ACC and amygdala are involved in the direct representation of the bodily state. Moreover, S1 and the insula are also related to emotion processing [13–16]. Researchers found that damage to the right S1 had subtle effects on emotional tasks and experiences [17, 18]. Brain regions associated with pain may interact with each other during the processing of pain information [19]. In response to this view, some studies have found that the integration of excitatory neurons in S1 originates from pain information from the peripheral nerves and is transmitted to other pain-related brain regions [20, 21]. Using the two-photon calcium ion imaging technology, it has been found that the spontaneous activity and sensory response in S1 and stimulation of S1 can increase chronic pain [22]. Inhibition of S1 activity can attenuate chronic pain. In addition, they found that the electrical response of the ACC to peripheral stimulation was consistent with the activity of S1 neurons. The inhibition of ACC activity can reduce the mechanical touch and pain, indicating that the excitatory neuronal activity in S1 increases responses to pain behavior by promoting activation of the ACC. Early studies on pain affection focused on psychological research; with the remarkable development of human imaging research, we have a deeper insight into the brain network which regulates pain and affection interaction. Pain perception and affection interact with each other. S1 and ACC are the main brain regions regulating pain and affection, respectively, so S1 and ACC may be closely correlated.

Electroacupuncture (EA) is an important treatment method developed based on improving traditional Chinese medicine. It has good analgesic effects and is widely used clinically [23, 24]. Related animal studies have shown that EA can increase the pain threshold and can effectively regulate pain-related emotion and cognitive behavior disorder [25, 26]. At the same time, in clinical research, EA not only alleviates various kinds of acute and chronic pain [27] but also significantly improves emotional symptoms [28]. However, whether the mechanism of the effect of EA on pain perception and pain-related affect is similar is still unclear.

Therefore, in the present study, we examined chronic inflammatory pain perception, pain-paired aversion, and pain-related anxiety in rats with and without an ACC lesion and S1 activated and synchronous neural oscillations in S1, to explore whether pain perception and negative affection influence each other based on ACC and/or S1, and if the effect of EA on chronic pain is a result of the effect of EA on negative affect.

## 2. Material and Methods

### 2.1. Animals and Groups

Seventy adult male Sprague-Dawley rats (Animal Experiment Center, Zhejiang Chinese Medical University, Zhejiang, China), weighing 250-280 g, were group-housed, with 3-4 rats per cage, in an environmentally controlled room (24-26°C, 40-50% humidity) and kept on a 12 h light-dark cycle with free access to rodent chow and water. The whole experiment was performed under the guidelines of the International Association for the Study of Pain and the Institutional Animal Ethical Committee (IAEC).

The 50 rats were randomly divided into a blank control group (control group), complete Freund's adjuvant- (CFA-) induced chronic pain model group (model group), model+EA group, model+ACC lesion group (ACC lesion group), and ACC lesion+EA group (ACC lesion+EA group). This part was to explore whether pain perception and negative affect influence each other based on ACC.

The other 20 rats were divided into the control-hM3D-saline (C-hM3D-saline group) and the control-hM3D-clozapine-N-oxide (C-hM3D-CNO group). This part was to explore whether pain perception and negative affect influence each other based on S1.

### 2.2. Surgeries

#### 2.2.1. ACC Lesion

The rats were anesthetized using urethane (1.2 g/kg i.p., Sigma-Aldrich, St. Louis, MO, USA), fixed to a stereotaxic apparatus (68025, RWD Life Science, China), and maintained at a constant body temperature of 37°C with real-time monitoring of temperature changes. After using iodine to disinfect the head of the rats, we cut the scalp and exposed the anterior fontanelle, posterior fontanelle, and frontal bone of the skull. A dental drill was used to drill holes in the surface of the rat skull, and one cranial nail was fixed on each side with dental cement. According to *The Rat Brain in Stereotaxic Coordinates* by Paxinos and Watson, the electrodes were slowly inserted into the ACC using a stereotaxic apparatus (+2.7 mm rostrocaudal, +1 mm mediolateral, and 2.0 mm dorsoventral). The biaxial electrode in the ACC was connected to the lesion-making device; 1 mA direct current was supplied for 60 s to create the lesion. After the surgery, the rats were rested for a week.

#### 2.2.2. S1 Electrode Implantation

To record the electrophysiological signals, we implanted array electrodes in S1. The preoperative procedure was the same as that for an ACC lesion surgery. The recording electrode array was positioned according to the rat brain atlas (-1.32 mm rostrocaudal, +2.5 mm mediolateral, and 2.35 mm dorsoventral).

### 2.3. Electroacupuncture Treatment

The model+EA and lesion+EA groups received EA intervention from the second day to the fourteenth day after model induction using the Master-9 electric pulse stimulator made in USA, seven times, every other day. The fine needle was 0.25 mm in diameter and 13 mm in length. The EA treatment was performed at bilateral Housanli acupoints and the reference electrode (1 cm inferior to the Housanli acupoint); the following stimulation parameters were used: frequency, 2/100 Hz; duration, 30 min; and intensity range, 0.5-1.5 mA (set at 0.5 mA initially and increased by 0.5 mA every 10 min). The other groups were given constraints as EA but not EA treatment.

### 2.4. Behavioral Tests

#### 2.4.1. Paw Withdrawal Thresholds

After the paw withdrawal threshold (PWT) was stable, the baseline PWT before model induction and the mechanical pain threshold of rats at 26 h and day 16 (16 d) after model induction were measured, excluding those with an abnormal pain threshold before the experiment (pain threshold < 10 g or >40 g). The measurement method was using a dynamic plantar tactile instrument (model 37450; Ugo Basile, Comerio, Italy) following a previously described method [29].

#### 2.4.2. Conditioned Place Aversion Testing

The CFA-induced pain-paired aversive behavior was tested using a modified conditioned place aversion (CPA) paradigm. Rats in each group were free to move and train in two equally sized cabinets (A and B, each sized 35 cm × 28 cm × 45 cm), with a removable and installable baffle between the two cabinets. The walls of the two cabinets were composed of wallpaper strips of varying widths (3 cm vs. 9 cm) and colors (black vs. white). The bottom of the apparatus was hollowed out and could be placed on a pain measuring rack. A camera was installed above the apparatus and connected to the animal video tracking system software to adjust the video picture to facilitate subsequent tracking and recording. The surrounding environment (temperature, 24-25°C; humidity, 40-60%; noise, <40 dB) before the experiment was controlled carefully, thus maintaining a quiet environment. The modified CPA paradigm was divided into three parts. First, on the free behavior day, the baffle between two cabinets was removed, and the rats were allowed to move freely in both cabinets for 30 min (a 1 min preparatory period was given and not included in the final data). The retention time of the rats spent in cabinet A and cabinet B was recorded. The conditional and nonconditional boxes of rats were randomly determined. Second, on the preconditioning day, the baffle was installed, and rats that had not undergone model induction were placed in the previously determined nonconditioned box for 30 min. Third, on the conditional day, the control group was injected with 50 *μ*l saline; the other four groups were injected with 50 *μ*l CFA. Two and twenty-six hours after model induction, the rats underwent mechanical plantar stimulation with a dynamic plantar tactile instrument (model 37450; Ugo Basile) as part of the conditioned training for 30 min. The rats were first placed in the conditioning cabinet for 5 min to adapt; the left foot of the rats was subjected to mechanical PWT testing (the methods and parameters were the same as the mechanical pain measurement) for 6-10 min. After PWT values were obtained, the control group was subjected to mechanical foot stimulation at (PWT × 0.5)/5 s (i.e., maximum stimulation is half of the PWT and is reached over 5 s). The number of times the rat lifted its foot during the 5 s period was recorded. Stimulation was performed once every min, and the number of foot lifts within 20 min was recorded. The mechanical pain threshold was measured using (PWT × 1.5)/5 s for the last 20 min in the other groups (i.e., the maximum stimulation was 1.5 times the PWT); the remaining procedures were performed the same as those in the control group. Fourth, on the second, ninth, and fifteenth postconditioning days after model induction, the rats were placed in open cabinets freely for 30 min. The activity time in each cabinet was recorded.

Aversive behavior was calculated using the CPA score, which is the difference in time between the test day (2 d, 9 d, and 15 d) and baseline for the pain-paired cabinet. The formula was as follows: *T*_score_ = *T*_preconditioning_ − *T*_postconditioning_.

At the beginning of the experiment, the rat was gently lifted by the tail and placed in the center of the two cabinets. After a 1 min acclimation period, the activity of the rat in the cabinets was monitored and observed using the Smart 3.0 software (Panlab, USA), and data were collected for 30 min. After each experiment, the apparatus was thoroughly scrubbed with 10% alcohol to eliminate feces and prevent residual odor from interfering with the activities of the next rat.

### 2.5. Local Field Potentials (LFPs)

LFPs in S1 were recorded during the free period before and 2 h, 2 d, 9 d, and 15 d after modeling, using the implanted array electrode with a Cerebus neural signal processing system (Blackrock Microsystems, Salt Lake City, UT, USA). Behavioral tasks and LFP signals were linked using the ANY-maze interface system (Stoelting, CO, USA) to synchronously record behavioral monitoring and LFP signals. The extracellular LFPs in S1 were recorded using the array microelectrode embedded in the rat S1 with the Cerebus 128 multichannel in vivo recording system (Blackrock Microsystems, Salt Lake City, UT, USA). The LFP signal was amplified using a preamplifier (×300), band-pass filtered at 0.3-250 Hz, and sampled at 1 kHz. After the signal acquisition, the LFP signal data collected from each channel in the S1 brain region were band-pass filtered and processed at 2-45 Hz through the NeuroExplorer 5.021 (NEX, Plexon Inc., USA) and MATLAB analysis software (MathWorks, Natick, MA, USA), and the average power spectral densities (PSD) of each group and the PSD percentage of each frequency band were compared.

### 2.6. Open Field Testing

A black Plexiglas chamber (100 cm × 100 cm × 50 cm) formed the apparatus, which was divided evenly into 16 small squares (25 × 25 cm/each). The four squares in the center were defined as the central areas and the other twelve as the peripheral areas. Before testing, the rats were put into the experimental environment for an hour to adapt to it. Then, the rats were gently placed into the central areas with their heads facing away from the experimenter. The behavior was videotaped for 5 min by the Smart 3.0 system (Panlab, USA). The whole apparatus was wiped with 75% ethanol before each trail.

### 2.7. Chemical Genetic Method

The preoperative procedure was the same as that for the S1 electrode implantation. Then, viruses were injected into S1, rAAV-hSyn-hM3D(Gq)-EGFP-WPRE-pA (virus titer: 2.79 × 10^12^ vg/ml, 60 nl/min, 350 nl/injection; BrainVTA, Wuhan, China). Thirty minutes before the behavioral assessment, the designer drug CNO (10 mg/kg, i.p.; C0832, Sigma-Aldrich, St. Louis, MO, USA) was administrated. The same volume of saline was administered to the C-hM3D-saline group.

### 2.8. Data Analysis

The experimental data are presented as the mean ± standard error of the mean. One-way or two-way repeated measures analysis of variance (rm-ANOVA) with the Bonferroni post hoc analysis was used when the variances were equal. One-way ANOVA was used for multigroup comparison, and the LSD test was used for two-to-two comparisons between groups. In the case of an uneven variance, Dunnett's T3 test was used for two-to-two comparisons between groups. Results were considered statistically significant at *P* < 0.05.

## 3. Results

### 3.1. Pain Perception

**Figure 1:**
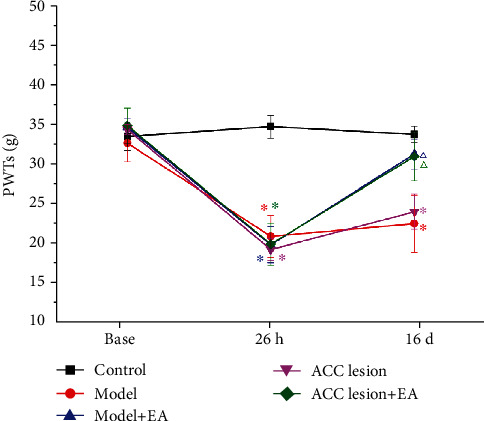
PWT of the ipsilateral (left) hind paw in each group. ^∗^*P* < 0.05 compared with the control group. ^△^*P* < 0.05 compared with the model group. *n* = 8. Abbreviations: PWT: paw withdrawal threshold; EA: electroacupuncture; ACC: anterior cingulate cortex.

As shown in Figure 1, there was no significant difference before CFA injection between the control, model, model+EA, ACC lesion, and ACC lesion+EA groups (two-way rm-ANOVA; *P* > 0.05). Compared with the control group 26 h after CFA injection, the PWTs significantly decreased in the other four groups (*P* < 0.05, Bonferroni test). On 16 d, compared with that in the control group, the PWTs in the model group and ACC lesion group were significantly lower (*P* < 0.05, Bonferroni test). Compared with that in the model group, the PWTs in the model+EA group and ACC lesion+EA group were significantly higher (*P* < 0.05, Bonferroni test) (Figure 1).

**Figure 2:**
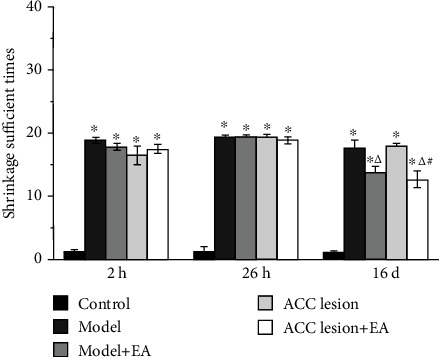
The shrinkage sufficient times for the left hind paw in each group. ^∗^*P* < 0.05 compared with the control group. ^△^*P* < 0.05 compared with the model group. ^#^*P* < 0.05 compared with the ACC lesion group. Abbreviations: EA: electroacupuncture; ACC: anterior cingulate cortex.

We also detected the lifted foot times of the rats' left foot after CFA injection. Compared with that in the control group, the lifted foot times in the four groups was significantly higher at 2 h, 26 h, and 16 d (*P* < 0.05, Bonferroni test). Compared with that in the model group at 16 d, the shrinkage sufficient times in the model+EA group and ACC lesion+EA group were significantly lower (*P* < 0.05, Bonferroni test). The ACC lesion+EA group had a significantly lower lifted foot times than the ACC lesion group (*P* < 0.01, Bonferroni test) (Figure 2).

**Figure 3:**
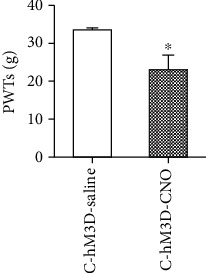
Paw withdrawal thresholds decreased by the specific activation of S1 glutaminergic neurons in the control rats. ^∗^*P* < 0.05 compared with the C-hM3D-saline group. Abbreviations: PWT: paw withdrawal threshold; S1: primary somatosensory cortex.

Then, we measured the PWTs in rats after the activation of S1 by the chemical genetic method. Compared with the C-hM3D-saline group, the PWTs in C-hM3D-CNO rats were significantly decreased (*P* < 0.05, Bonferroni test) (Figure 3).

### 3.2. Pain-Related Emotional Behavior

#### 3.2.1. Pain-Related Emotional Behavior after an ACC Lesion

**Figure 4:**
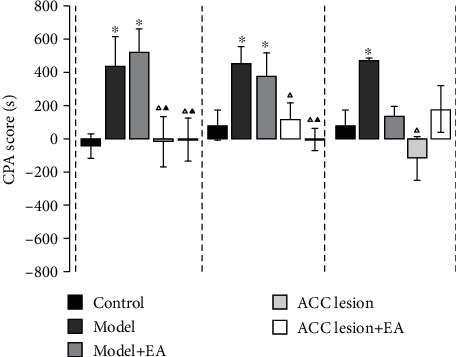
Values of aversive behavioral testing. ^∗^*P* < 0.05 compared with the control group. ^△^*P* < 0.05 compared with the model group. ^▲^*P* < 0.05 compared with the model+EA group. Abbreviations: CPA: conditioned place aversion; EA: electroacupuncture; ACC: anterior cingulate cortex.

The experimental results showed that there was no significant difference between the five groups of the rats in the conditioning cabinet or nonconditioning cabinet before the injection of CFA. On 2 d, the CPA scores in the model and model+EA groups were significantly higher than those in the control group (*P* < 0.05, Bonferroni test). Compared with the CPA score in the model group, the ACC lesion group and ACC lesion+EA group had significantly lower values (*P* < 0.05, Bonferroni test). Nine days after CFA injection, compared with that in the control group, the CPA score in the model and model+EA groups was significantly higher (*P* < 0.05, Bonferroni test); compared with the model group, the ACC lesion and ACC lesion+EA groups had significantly lower scores (*P* < 0.05, Bonferroni test); compared with the model+EA group, the ACC lesion+EA group had significantly lower scores (*P* < 0.01). Fifteen days after CFA injection, compared with the CPA score in the control group, the model group had a significantly higher score (*P* < 0.05, Bonferroni test), but the model+EA group showed no significant difference. Compared with the model group, the ACC lesion group had a significantly lower score (*P* < 0.01, Bonferroni test). There was no significant difference in the score between the model+EA group and ACC lesion+EA group (Figure 4).

#### 3.2.2. Pain-Related Emotional Behavior after S1 Activation

**Figure 5:**
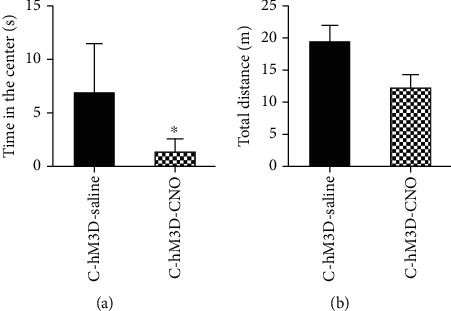
Anxiety disorders induced by the specific activation of S1 glutaminergic neurons in control rats. (a) Time in the center. (b) Total distance. ^∗^*P* < 0.05 compared with C-hM3D-saline group. Abbreviations: C: control; CNO: clozapine-N-oxide.

The results showed that the time in the center of the C-hM3D-CNO rats was significantly increased in the open field testing (OFT) (*P* < 0.05, Bonferroni test), compared with the C-hM3D-saline group (Figure 5).

### 3.3. LFP Signals in S1

**Figure 6:**
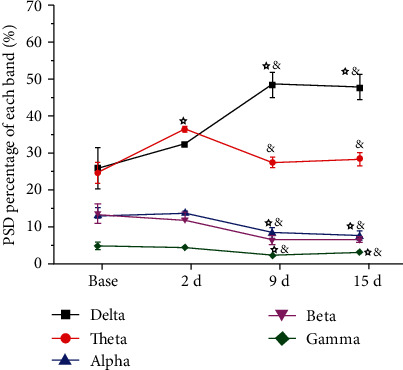
Synchronous LFPs observed during nociceptive behavioral testing in the model group. ^☆^*P* < 0.05 compared with the baseline. *^&^P* < 0.05 compared with 2 d. Abbreviations: PSD: power spectral density; LFP: local field potential.

Different frequency bands in the S1 brain activity of the model group changed at different time points relative to those in the model group. Compared with the baseline, the delta frequency PSD was significantly higher at 2 d, 9 d, and 15 d (one-way rm-ANOVA, *P* < 0.01). Compared with the baseline value, the theta band PSD two days after CFA injection was significantly higher (one-way rm-ANOVA, *P* < 0.05); PSDs at 9 d and 15 d were significantly lower than those at 2 d (one-way rm-ANOVA, *P* < 0.05). Compared with the values at baseline and 2 d, the PSDs of the alpha, beta, and gamma bands were significantly lower at 9 d and 15 d (one-way rm-ANOVA, *P* < 0.05) (Figure 6).

**Figure 7:**
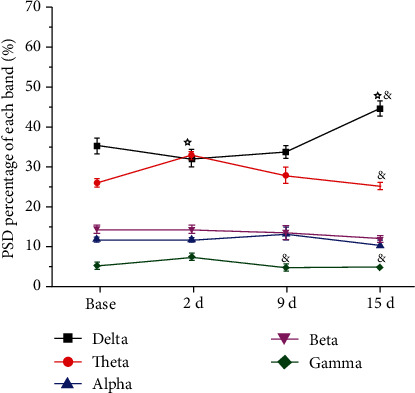
Synchronous LFPs observed during nociceptive behavioral testing in the model+EA group. ^☆^*P* < 0.05 compared with the baseline. ^&^*P* < 0.05 compared with 2 d. Five frequency band intervals were considered: delta (2-4 Hz), theta (4-9 Hz), alpha (9-15 Hz), beta (15-30 Hz), and gamma (30-45 Hz). Abbreviations: PSD: power spectral density; LFP: local field potential.

In the model+EA group, the delta band in the S1 brain region at different time points was significantly different. Compared with that at the baseline, the delta band PSD was significantly lower at 2 d (one-way rm-ANOVA, *P* < 0.05). Compared with that at 2 d, the PSD of the delta band was significantly higher at 15 d (one-way rm-ANOVA, *P* < 0.05). For the theta band, compared with that at the baseline, the PSD was significantly higher at 2 d (one-way rm-ANOVA, *P* < 0.05); the value at 15 d was significantly lower than that at 2 d (one-way rm-ANOVA, *P* < 0.05). The gamma band PSD was significantly lower at 9 d and 15 d compared with that at 2 d (one-way rm-ANOVA, *P* < 0.05). In addition, the alpha and beta bands were not significantly different at any of the time points (Figure 7).

**Figure 8:**
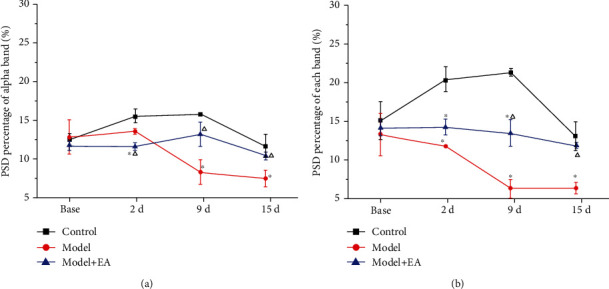
The effect of EA on the PSD percentage of the alpha (a) and beta (b) bands in the S1 of CFA rats. ^∗^*P* < 0.05 compared with the control group. ^△^*P* < 0.05 compared with the model group. Abbreviations: PSD: power spectral density; EA: electroacupuncture; S1: primary somatosensory cortex; CFA: complete Freund's adjuvant.

Furthermore, we analyzed the alpha and beta bands for changes in the S1 brain region at different times. There was no significant difference in the PSD of each band at the baseline. Compared with that in the control group, the PSD of the alpha band in the model+EA group was significantly lower at 2 d (one-way rm-ANOVA, *P* < 0.01), and the PSD of the model group was significantly lower at 9 d and 15 d after CFA injection (one-way rm-ANOVA, *P* < 0.05). Compared with that in the model group, the alpha band PSD in the model+EA group was significantly higher at 9 d and 15 d (one-way rm-ANOVA, *P* < 0.05) (Figure 8(a)).

Compared with that in the control group, the PSD percentage of the beta band in the model group and model+EA group was significantly lower at 2 d, 9 d, and 15 d after CFA injection (one-way rm-ANOVA *P* < 0.01). Compared with that in the model group, the PSD percentage of the beta band in the model+EA group was significantly higher at 9 d and 15 d (one-way rm-ANOVA, *P* < 0.01) (Figure 8(b)).

## 4. Discussion

The results showed that ACC lesions have no effect on pain perception, but they relieve pain-paired aversion. EA can mitigate pain perception, regardless of the presence or absence of an ACC lesion. However, ACC lesions and EA have a similar effect on pain-related aversion. S1 activation induced lower pain threshold and anxiety disorder. CFA-induced chronic pain may increase the delta and theta band oscillatory activity in S1 and decrease the oscillatory activity of the alpha, beta, and gamma bands. EA intervention may inhibit the oscillatory activity of the alpha and beta bands. These results suggest that ACC is involved in the dissociation of pain perception and pain-related affection, while S1 may be related to the interaction of pain perception and pain-related negative affection. EA may improve pain-related aversion by influencing ACC activity and regulating theta power in S1.

### 4.1. ACC Lesion Induced Changes in Pain-Related Aversion in CFA Rats

ACC is a highly heterogeneous cortical region that connects the lateral and medial pain pathways, providing a physiological basis for the interaction between pain and affect [30]. Studies have found that synaptic transmission and plasticity in ACC play a vital role in pain, fear, learning, and memory. Joshua et al. were the first to propose the use of conditioned position aversion to study behavioral responses to pain-related emotions and successfully established the formalin-induced CPA (F-CPA) model, laying a foundation for studying the mechanism of negative emotions caused by pain [31]. Subsequently, Joshua et al. showed, for the first time, that rostral ACC (rACC) damage can weaken CPA. Researchers [32] further proved that the ACC was a brain region closely linked to pain-related emotions. It has been found that the long-term enhancement of ACC activity may be one of the mechanisms of persistent emotional change in patients with chronic pain caused by nerve injury or peripheral inflammation [33–36]. Our data showed that the PWT of the model group was significantly lower than that of the control group at 16 d. The group with the ACC lesion did not differ from the control group at 16 d. However, the pain-related aversion developed from 2 d to 16 d, and there was no manifestation of aversion after ACC lesioning. This suggests that pain perception and pain-related aversion are separate process in some specific brain areas, and ACC plays a key role in pain-related aversion.

Notably, pain-related aversion could be triggered easily and persisted for 16 days in this study. A prior study reported that neural activity in the ACC correlated with noxious intensities, and modulation of ACC neurons can regulate the aversive response to acute pain [37]. The manifestation of aversion enhances the progress of the primary disease and chronicity. It is necessary to inhibit the earlier pain-paired aversion behavior by controlling ACC activity.

### 4.2. After EA, ACC Lesion Induced Changes in Pain Aversion in CFA Rats

Compared with that in the model group, the PWT of the EA group and ACC lesion+EA group was significantly higher at 16 d; however, the ACC lesion showed no significant difference. This means that EA can relieve pain perception, and the effect is not the result of the ACC activity. The aversive behavior was improved in the EA, ACC lesion, and ACC lesion+EA groups relative to that in the model group. It suggests that EA can improve pain-related aversion, and the effect is similar in the presence of an ACC lesion. Remarkably, the effect of EA on pain-paired aversion gradually increased from 9 d to 16 d; thus, the cumulative effect of EA on pain-paired aversion is pivotal. EA can effectively relieve the negative emotions associated with pain, in which the ACC may be involved in the EA effect.

The effect in the ACC lesion group was different from that in the ACC lesion+EA group at 9 d. This suggests that EA may regulate pain-paired aversion through other mechanisms barring the ACC activity. As a higher structure, the ACC may integrate affective signals directly or indirectly through the projection of other regions, such as S1, the amygdala, insular cortex, medial prefrontal cortex, and hippocampus, among others [38]. The ACC has strong connections to multiple brain regions, which overlap with the regions that regulate pain [39]. The extensive connections prove that an interaction exists between pain perception and pain-related aversion. Human clinical studies have found [40] that surgical removal of ACC not only alleviates various pain-related feelings but also significantly alleviates depression, anxiety, and other emotions caused by pain in patients. According to our results, we did not find an influence of ACC lesions on pain perception, which demonstrates that pain perception and pain-paired aversion are separate processes.

### 4.3. Changes in S1 Neural Oscillations in Model Rats and after EA Intervention

Pain is a perception that is affected by an immense brain regional network, which involves multiple brain regions. The coding of various information, such as cognition, memory, and emotion, by the brain requires transmission and processing in multiple brain regions, while the neural oscillation produced by the brain can closely associate the activity of neurons in each brain region, which can strengthen interregional synergistic operations and information processing efficiency. It has been discovered that neural oscillation is associated with five frequency bands, comprising delta (2-4 Hz), theta (4-9 Hz), alpha (9-15 Hz), beta (15-30 Hz), and gamma (30-45 Hz).

In this study, there was no significant difference in the neural oscillation between various frequency bands in S1 before model induction between the groups. However, on the second day after constructing the chronic pain model using CFA injection, the PSD of the theta frequency band was remarkably enhanced, while that of the beta and gamma frequency bands was markedly reduced. On the ninth day after model induction, the PSD of the delta frequency band increased to a peak, while that of the alpha, beta, and gamma frequency bands decreased. This finding suggests that the delta frequency band might not be involved in the early formation of pain perception but gradually increases during the development of chronic pain. The PSD of the theta frequency band was dramatically enhanced immediately after the second day of model induction, which was gradually reduced to the level before model induction at 9 d and 15 d after model induction, indicating that the theta frequency band might participate in related information processing during the early formation of pain perception; however, the influence of the theta frequency band gradually reduced with the development of chronic inflammatory pain. Moreover, the PSD of the alpha, beta, and gamma frequency bands showed an apparent decreasing trend after model induction, demonstrating that the reduction in rhythmic oscillation activity of these three frequency bands in S1 was closely correlated with the formation of chronic pain.

It has been reported that reinforcement learning and feedback locking reward motivation are related to specific activity in the delta frequency band [41]. The theta frequency band is generally regarded as the neural oscillation frequency band that is related to pain perception, cognition, and memory [40, 42, 43]. The alpha frequency band is related to spontaneous and top-down visual-spatial allocation, which is markedly enhanced when attention is completely transferred but is gradually reduced when attention is balanced across the field of view [44]. The beta frequency band plays a distinct role during the perception process, and a marked reduction in the beta frequency band energy can be detected during the brain activity of perception conversion [45]. The gamma frequency band is also closely related to pain [46]. In our results, EA could reverse the decrease of PSD in the alpha and beta bands. Some studies have reported that the effect of EA on chronic pain is through regulating pain perception and pain-paired aversion.

The effect of EA in treating various pain-related diseases has been extensively recognized, and its analgesic effect can last for a long time after treatment. Neuroimaging research has indicated that the application of acupuncture or EA may induce extensive [47, 48] that mainly participate in the overlapping neural networks of pain transmission and perception. Moreover, acupuncture can also directly affect electroencephalographic activity in both healthy human populations and animals [49, 50]. EA intervention can reduce the amplitude of high-frequency band activity in rats with postoperative pain (especially for that of the beta frequency band) and reverse the increased strength of coupling of the crossover frequency between the beta and low-frequency band [51], demonstrating that EA may exert analgesia by regulating rhythmic intracerebral neural oscillation.

## 5. Conclusion

Our research reveals that ACC is involved in the dissociation of pain perception and pain-related affection, while S1 may be related to the interaction of pain perception and pain-related negative affection. EA may improve pain-related affection by influencing the ACC activity and regulating theta power in S1. These results suggest that EA may mitigate chronic pain by relieving pain perception and improving pain-related affection through different mechanisms. This evidence builds upon findings from previous studies of chronic pain and EA treatment.

## Data Availability

The figure types of the data used to support the findings of this study are included within the supplementary information file(s).
